# 3D Compressed Convolutional Neural Network Differentiates Neuromyelitis Optical Spectrum Disorders From Multiple Sclerosis Using Automated White Matter Hyperintensities Segmentations

**DOI:** 10.3389/fphys.2020.612928

**Published:** 2020-12-23

**Authors:** Zhuo Wang, Zhezhou Yu, Yao Wang, Huimao Zhang, Yishan Luo, Lin Shi, Yan Wang, Chunjie Guo

**Affiliations:** ^1^Key Laboratory of Symbol Computation & Knowledge Engineering, Ministry of Education, College of Computer Science & Technology, Jilin University, Changchun, China; ^2^Department of Radiology, the First Hospital of Jilin University, Changchun, China; ^3^Jilin Provincial Key Laboratory for Medical imaging, Changchun, China; ^4^BrainNow Research Institute, Hong Kong, China; ^5^Department of Imaging and Interventional Radiology, Chinese University of Hong Kong, Hong Kong, China

**Keywords:** Neuromyelitis optical spectrum disorder (NMOSD), multiple sclerosis (MS), magnetic resonance imaging (MRI), deep learning, convolutional neural networks (CNNs)

## Abstract

**Background:**

Magnetic resonance imaging (MRI) has a wide range of applications in medical imaging. Recently, studies based on deep learning algorithms have demonstrated powerful processing capabilities for medical imaging data. Previous studies have mostly focused on common diseases that usually have large scales of datasets and centralized the lesions in the brain. In this paper, we used deep learning models to process MRI images to differentiate the rare neuromyelitis optical spectrum disorder (NMOSD) from multiple sclerosis (MS) automatically, which are characterized by scattered and overlapping lesions.

**Methods:**

We proposed a novel model structure to capture 3D MRI images’ essential information and converted them into lower dimensions. To empirically prove the efficiency of our model, firstly, we used a conventional 3-dimensional (3D) model to classify the T2-weighted fluid-attenuated inversion recovery (T2-FLAIR) images and proved that the traditional 3D convolutional neural network (CNN) models lack the learning capacity to distinguish between NMOSD and MS. Then, we compressed the 3D T2-FLAIR images by a two-view compression block to apply two different depths (18 and 34 layers) of 2D models for disease diagnosis and also applied transfer learning by pre-training our model on ImageNet dataset.

**Results:**

We found that our models possess superior performance when our models were pre-trained on ImageNet dataset, in which the models’ average accuracies of 34 layers model and 18 layers model were 0.75 and 0.725, sensitivities were 0.707 and 0.708, and specificities were 0.759 and 0.719, respectively. Meanwhile, the traditional 3D CNN models lacked the learning capacity to distinguish between NMOSD and MS.

**Conclusion:**

The novel CNN model we proposed could automatically differentiate the rare NMOSD from MS, especially, our model showed better performance than traditional3D CNN models. It indicated that our 3D compressed CNN models are applicable in handling diseases with small-scale datasets and possess overlapping and scattered lesions.

## Introduction

Neuromyelitis optical spectrum disorder (NMOSD) is a rare aquaporin-4 immunoglobin G antibody (AQP4-IgG) mediated chronic disorder of the brain and spinal cord (Wingerchuk et al., 2007, 2015). Traditionally considered a subtype of multiple sclerosis (MS), NMOSD has been recognized as a distinct clinical entity based on unique immunologic features in recent years (Wingerchuk et al., 2015). Up to 70% of NMOSD patients have brain lesions visible on magnetic resonance imaging (MRI) (Kim et al., 2015). But only about half of NMOSD patients have typical brain lesions, and their distributions of NMOSD and MS are overlapped (Cacciaguerra et al., 2019). Furthermore, compared to MS and stroke, it is challenging to segment and quantify white matter lesions (WMLs) on T2-weighted fluid-attenuated inversion recovery (T2-FLAIR) images in NMOSD, as its lesions are often located very close to the ventricles. However, it is vital to differentiate NMOSD from MS. Some MS treatments such as β-interferon can worsen NMOSD (Jacob et al., 2012; Kim et al., 2012), but distinguishing between the two disease entities is challenging. Studies based on machine learning to discriminate NMOSD from MS are limited.

Machine learning algorithms that precede human observation have shown application potential in medical image processing (Wernick et al., 2010). These algorithms handle a large number of features extracted from patients and lack inconsistencies (Eshaghi et al., 2016). Therefore, machine learning algorithms can build decision systems to support the diagnostic process. Previous studies have proved the efficiency and robustness of machine learning algorithms for many common diseases, such as breast cancer (Rastghalam and Pourghassem, 2016), brain tumors (Zacharaki et al., 2009), etc. However, NMOSD is a rare disease, which is a lack of large-scale public datasets for scientific research, and its similar phenotypes with MS bring challenges to build high-performance machine learning models. Laura Cacciaguerra et al. used typical/atypical brain and spinal cord lesions to construct a possible evidence-based diagnostic machine learning algorithm to discriminate NMOSD from MS with the sensitivity of 0.92, 0.82, and specificity of 0.91,0.91 in training and validation samples separately. However, the blinded machine learning approaches were not conducted yet (Cacciaguerra et al., 2019). Eshaghi et al. built a machine learning classifier using brain gray matter (GM) imaging measures to distinguish patients with MS from those with NMOSD with an average accuracy of 74%. When they used thalamic volume together with the white matter lesion volume, the classifier achieved an average accuracy of 80% (Eshaghi et al., 2016). Machine learning-based models indeed show applicational potential for medical image processing; however, there are some issues to resolve. First, medical images can’t be the model’s input directly; all features have to be extracted from the raw images by radiologists, which means the radiologists have already studied the raw data’s intrinsic information. Feature extraction processing, which is the bottleneck of the models’ performances, is highly influenced by the radiologists’ subjective judgments. Second, manual intervention is indispensable for both the training and testing phases, which means radiologists must process all images.

As a subfield of machine learning, deep learning can solve the problems as mentioned above. Deep learning algorithms can efficiently extract raw images’ features through convolutional neural networks (CNNs), and they are also widely applied to the medical images’ classification, segmentation, and detection tasks (Litjens et al., 2017). Researchers have conducted on the deep CNNs and have achieved better results than with other machine learning algorithms. Shen et al. (2015) proposed M-CNN to distinguish between malignant and benign nodules without nodule segmentation. Nie et al. (2016) proposed a three-dimensional (3D) CNN model to predict the overall survival (OS) for brain glioma patients. Payan et al. used 3D-CNN to process brain MRI data of Alzheimer’s disease (AD), and the classification of AD reached an accuracy rate of 89.47% (Payan and Montana, 2015). Wang et al. built an ensemble 3D-DenseNet to predict AD (Wang H. et al., 2019). U-net, proposed by Ronneberger et al. (2015), had a good effect on two-dimensional (2D) medical image segmentation. Based on this, Milletari et al. (2016) combined U-net and ResNet to propose V-net to solve the image segmentation problems of 3D data. Previous work based on 3D deep learning models focused on diseases with large data sets and concentrated lesions, such as AD and tumors. Multiple 3D-CNNs were used to extract the features and demonstrated the effectiveness of dealing with 3D images. 3D medical images are the input of 3D CNN models, which can reflect the whole lesion. However, there is still a lack of 3D CNN models to handle rare diseases with small-scale datasets, such as NMOSD. Significantly, the lesion distributions of NMOSD and MS are overlapped, which brings more challenges to build a high-performance 3D CNN model. 2D CNN model with less learnable parameters is more easily trained with small-scale datasets. Our experiment created a new model combining the advantages of 3D and 2D CNN models to differentiate NMOSD from MS in terms of WMLs segmentation with less learnable parameters and achieved better performance than the 3D ResNet baseline.

In this paper, we (i) investigated the traditional 3D CNN model for a 3D MRI data classification task and found that the conventional approach lacks generalization ability for NMOSD and MS classification; (ii) presented a two-view 2D model to boost the classification performance, comparing models that were pre-trained on an ImageNet dataset with models not pre-trained; (iii) set up experiments to analyze the primary factors influencing the experiments’ results.

## Materials and Methods

### Data Description

#### Participants

A retrospective sample of 41 NMOSD patients diagnosed according to the revised 2015 diagnostic criteria (Wingerchuk et al., 2015) was recruited in this work. 47 MS patients who had received their diagnosis according to the 2010 McDonald Criteria (Polman et al., 2011) were enrolled, and they also fulfilled the recently revised diagnostic criteria (Thompson et al., 2018). The MS group matched for age, sex, disease duration, and Expanded Disability Status Scale (EDSS) (Kurtzke, 1983) scores to the NMOSD group. All patients in the acute disease phase with brain MRI lesions were included in this study. Clinical characteristics, including EDSS scores of all patients, were performed within 48 hours from the MRI acquisition (Table 1). All the patients undergoing high-dose corticosteroid treatment or with a medical condition that could result in hyperintensity on T2-weighted and T2-FLAIR images were excluded. Besides, neurological comorbidities, a history of head trauma or surgery, and low-quality images or severe motion artifacts were excluded. This study was approved by the local ethics committee, and written informed consent was obtained from all participants.

**TABLE 1:** Demographic, clinical characteristics and brain WMH volume measurement.

	**MS (*n* = 47)**	**NMOSD (*n* = 41)**	***P*-value**
Age (years)	40.0 ± 11.1	44 ± 13	0.0865
Gender	14 M/33F	8 M/33 F	0.2720
Disease Duration (months)	57.1 ± 73.4	35.0 ± 42.9	0.1133
EDSS	3.6 ± 1.8	4.2 ± 2.4	0.1949
ICV (ml)	1407.2 ± 171.2	1367.3 ± 120.3	0.2165
WMH (ml)	9.0 ± 8.8	3.5 ± 4.8	0.0007
Normalized WMH (% of ICV)	0.66 ± 0.65	0.26 ± 0.37	0.0008

#### MRI Acquisition

The MRI protocol included 3D T2-FLAIR and 3D T1-weighted (T1W) fast field echo (FFE) sequences were obtained from the same 3.0T Philips Ingenia scanner (Philips Healthcare, Best, Netherland) between July 2015 and April 2018. There were some acquisition parameter variations over the years, where images were acquired axially or sagittally with parameter ranges. Sagittal/Axial T2-FLAIR: repetition time (TR) = 4800 m sec, echo time (TE) = 279–324 m sec, flip angle = 90°, number of slices = 160–192, field of view (FOV) = 220 mm, acquisition matrix = 224^∗^224, section thickness = 2 mm. Sagittal T1W: repetition time (TR) = 7.0–7.8 m sec, echo time (TE) = 3.2–3.6 m sec, flip angle = 7°, number of sections = 160–192, field of view (FOV) = 220 mm, acquisition matrix = 240^∗^240, section thickness = 1 mm.

### Automated Segmentation

**FIGURE 1:**
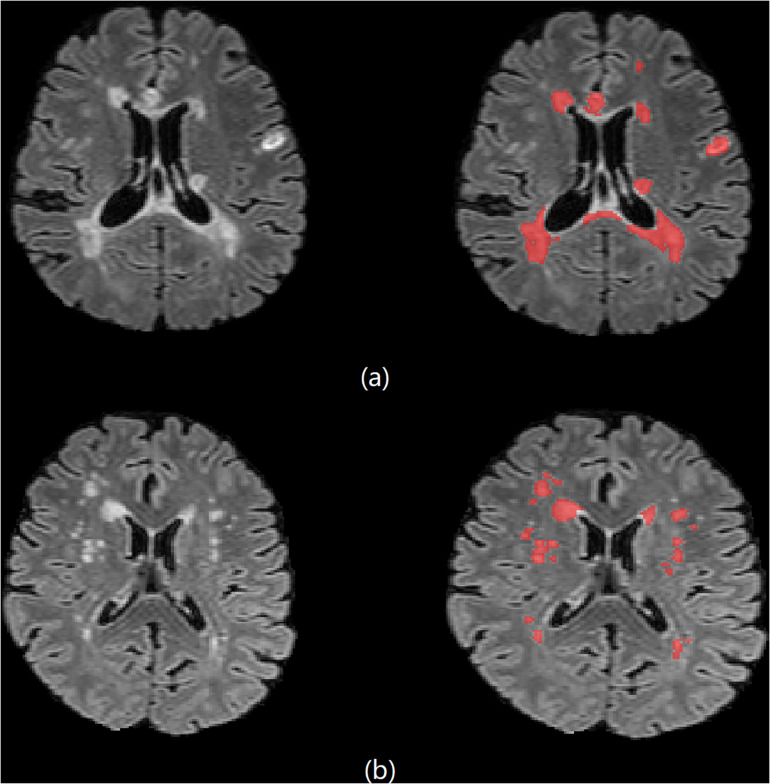
Brain 3D T2-FLAIR MR images of **(a)** one 33-year-old female MS patient and **(b)** one 61-year-old female NMOSD patient.

The automated segmentation software, AccuBrain IV1.0^®^ (Luo, 2017; Abrigo et al., 2019; Guo et al., 2019; Wang C. et al., 2019) (BrainNow Medical Technology Limited, Hong Kong, China), was used in the WML segmentation. Using AccuBrain, WML segmentation was automatically performed on T1W and T2-FLAIR images. Segmentation was first performed on T1 images, where brain structure masks and tissue masks were generated. Then, T1W and T2-FLAIR images were co-registered, and the structure and tissue masks were transformed into the T2-FLAIR space. Using a coarse-to-fine white matter hyperintensities (WMH) segmentation process, which utilizes mathematical morphological operations including binary dilation, grayscale closing, binary reconstruction, and grayscale reconstruction (Shi et al., 2013), WMH is extracted on T2-FLAIR images and is refined using the transformed brain structure mask from T1WI. The intracranial volume (ICV) is also calculated automatically, then the normalized WMH (% of ICV) is available. Figure 1 shows examples of the results of automated segmentation. The performance of WMH has been validated to have good accuracy and reproducibility (Guo et al., 2019).

### Data Processing

**FIGURE 2:**
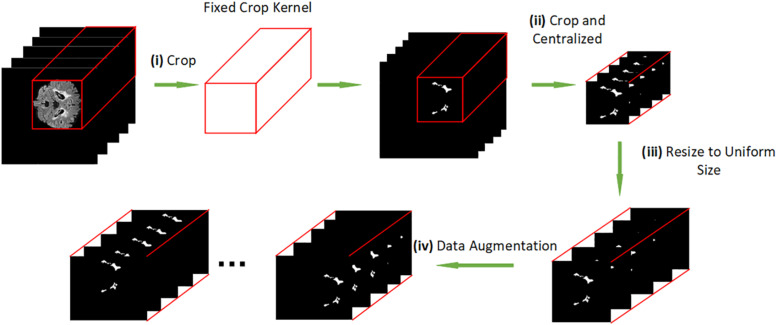
**(i)** cropping the raw MRI T2-FLAIR images to remove the background. **(ii)** cropping the brain lesion image with the same shape as the resized brain MRI T2-FLAIR images. **(iii)** resizing each slice of peer MRI T2-FLAIR images to 100*100*100 pixels. **(iv)** data augmentation.

Since NMOSD is a rare disease, it hard to find large-scale MRI datasets for research. We have collected the MRI datasets with a wide time range, so there are small differences in the size of images. Hence, we unified and centralized the raw images for the first time. The diagram of our data preprocessing procedure is shown in Figure 2. The method is carried out in three steps: i) we crop the raw MRI T2-FLAIR images to remove the black background and get the fixed crop kernel; ii) we crop the WMH brain lesion image with the fixed crop kernel that obtained at pervious step. iii) we use the OpenCV package to resize each section of peer MRI data to unify the data shape to 100^∗^100^∗^100 pixels; (iv) we apply data augmentation by shifting and flipping.

### Data and Code Availability Statement

The reconstruction algorithms to support the findings of this study are still under early-stage development. For the datasets, we do not share them directly due to the ethics of clinical research. Both the codes and data can only be acquired via a special request to the corresponding author.

## Model Structure

Previous studies have pointed out that the deeper the network, the stronger its learning ability (Bengio and Lecun, 2007; Bianchini and Scarselli, 2014; Montufar et al., 2014). But information loss as a common problem of traditional deep networks often appears in the process of information transmission. At the same time, the model is hard to train because of the gradient vanishing and the gradient explosion. He K. et al. proposed the idea of residual learning to solve this problem: To maintain the integrity of the information, He K. et al. proposed a model structure to bypass the input information to the output, which makes the model deeper (He et al., 2016). Formally, we consider a ResNet block defined as:

(1) y=F⁢(x,θ)+x

where *x* and *y* are the input and output of the ResNet blocks considered. θ is a set of learning parameters of function *F*(*x*).

Batch normalization (BN) is added to help the network increase generalization ability and accelerate the training process (Ioffe and Szegedy, 2015). ReLU is added to the network as a non-linear activation function.

### 3D ResNet for Image Classification

In this section, inspired by the work of Hara, K. et al. and ResNet (He et al., 2016), and compared with the previous studies of 3D CNN models (Payan and Montana, 2015; Hara et al., 2017, 2018; Liang et al., 2018; Wang H. et al., 2019), we used 3D ResNet as baseline models to solve the classification problem of NMOSD and MS MRI T2-FLAIR images. For grayscale MRI T2-FLAIR images, the shape of input data for the network is (1,*h*,*w*,*l*), and (*h*,*w*,*l*) representing the shape size (height, width, length) of each MRI T2-FLAIR image. The 3d convolution layer with input size(*C*_*in*_,*h*_*in*_,*w*_*in*_,*l*_*in*_) and output (*C*_*out*_,*h*_*out*_,*w*_*out*_,*l*_*out*_) can be precisely described as:

(2) $$o\,u\,t\,\left(C_{o\,u\,t}\right)=\sum_{k=0}^{C_{i\,n}-1}w\,e\,i\,g\,h\,t\,\left(k\right)\,i\,n\,p\,u\,t\,\left(k\right)+b\,a\,i\,s$$

where is the valid 3D cross-correlation operator, and 2d convolution calculates similarly.

### 2D ResNet for Image Classification

**FIGURE 3:**
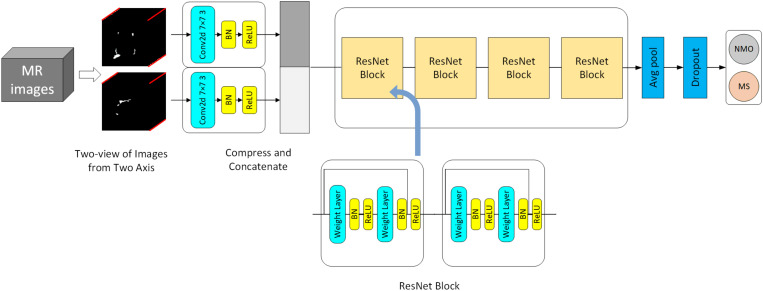
2D model architecture for classification of NMOSD and MS.

The large sample space and small data sets bring challenges to the 3D deep learning model’s training. Transfer learning can improve the model (Pan and Yang, 2010; Tajbakhsh et al., 2016), but there is no available 3D pre-training model for image classification. On the other hand, it’s impossible to distinguish NMOSD from MS by a single 2D slice because of the highly overlapping brain lesions. A compression block proposed to extract the most critical features each 2D slice by one view and map the 3D input (1,*h*,*w*,*l*) to a 2D form (*c*,*h*′,*w*′), where *c* represents the image’s channel. The convolutional kernels calculate each channel and sum them up to new channels that merge each 2D slice’s internal relationship, making the mapping operation lose structural information. Therefore, we proposed a two-view structure to extract the essential features from two different axes. The internal structure information is retained by concatenating the compression blocks’ output, which effectively reduces the sample space and can apply the compressed data to transfer learning. Figure 3 shows our model architecture, and the compression block used in our model. In the compression block, the first convolution layer is designed to extract information from input image. After many attempts with different parameters, we set the convolution layer with a kernel size of 7 and stride of 3, and the output channel of this layer is 32, which may get the best performance. The compression operation can be precisely described as:

(3) $$x\,\left(h,w,l\right)\,\overset{h\to c}{\Rightarrow }x_{1}\,\left(c,w,l\right)$$

(4) $$x\,\left(h,w,l\right)\,\overset{w\to c}{\Rightarrow }x_{2}\,\left(h,c,l\right)$$

(5) $$y_{1}=\sigma \,\left(B\,N\,\left(c\,o\,n\,v\,2\,d\,\left(x_{1}\right)+b_{1}\right)\right)$$

(6) $$y_{2}=\sigma \,\left(B\,N\,\left(c\,o\,n\,v\,2\,d\,\left(x_{2}\right)+b_{2}\right)\right)$$

(7) $$y=c\,o\,n\,c\,a\,t\,e\,n\,a\,t\,e\,\left(y_{1},y_{2}\right)$$

where BN is the batch normalization layer, and σ denotes ReLU. Equation (3), (4) demonstrates that we change one axis of 3D data to a channel, which means the compression block extract features from one view.

### Implementation Details

Our model is based on python and PyTorch. The optimizer used in training is SGD, the initial learning rate is 0.01, and the learning rate is reduced by 10% every ten steps. We added dropout before the final fully connected layer (Srivastava et al., 2014) to prevent model overfitting on the training data, with a dropout rate of 0.15. We trained our model on a server with one NVIDIA 1080ti. We also applied data augmentation for T2-FLAIR MRI images by shifting. To reduce the accidental factors, we conducted five-fold cross-validations to ensure that each image was tested at least once and also repeated the cross-validations process for 15 times to average the results. T2-FLAIR MRI images of 41 NMOSD subjects and 47 MS subjects were used for the experiments. The original data set was randomly divided into five equal-sized groups to utilize a five-fold cross-validation method to evaluate the model’s performance, which means four groups were used for model training, and one group was regarded as the validation dataset.

## Results

### 3D and 2D ResNet Model for Image Classification

**FIGURE 4:**
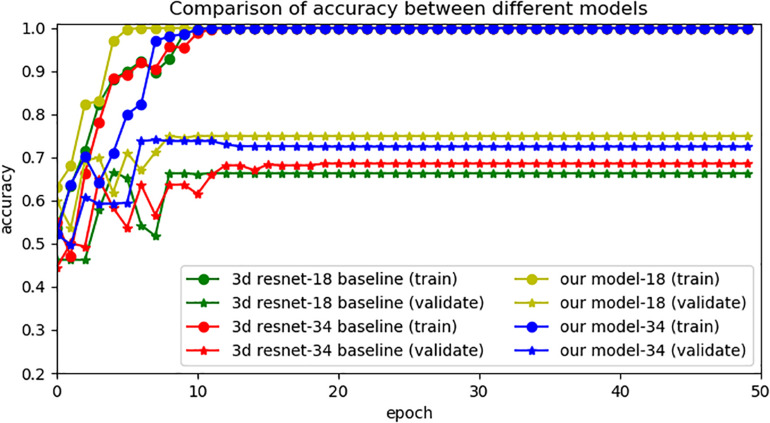
Comparison of accuracy between different models.

In this experiment, we applied a 3D RseNet model to process NMOSD and MS MRI T2-FLAIR images. The 3D model we used was based on the models proposed by Payan and Montana (2015), Hara et al. (2017, 2018), Wang H. et al. (2019), which were applied traditional 3D ResNet for classification tasks and achieved desired results. However, the overlapping lesions and the limited data set’s size of NMOSD and MS restrict the learning efficiency of 3D ResNet. Considering the above problems, we proposed a compression block to map the high dimensional 3D data into a lower dimension. It can also extract the information from the overlapping lesion locations by learning each single MRI section and building long-range dependence at the same time. After compressing the 3D data, we applied 2D ResNet for model processing and used transfer learning to improve the model’s generalization ability effectively. Figure 4 shows the accuracy of different models. We set 3D ResNet-18 (18 layers) and 3D ResNet-34 (34 layers) to the baseline models (Payan and Montana, 2015; Hara et al., 2017, 2018; Wang H. et al., 2019). This experiment demonstrates the limitations of traditional 3D CNN models’ learning capacity on NMOSD and MS datasets. The resulting diagram also showed that the accuracy of models changed rapidly, indicating the convergences in the training phase. In contrast, the validation phase’s unsatisfactory validation performances of baseline model showed that the generalization ability of the 3D ResNet was not ideal.

Overall, the experimental results indicated that our models have higher prediction performance, as embodied in higher validation accuracy. It also reflected that our model needs less time to train. The best performance was achieved when the model was pretrained on ImageNet datasets.

### Comparing Different Models

To compare the differences in performance between different models, we repeated the experiment 15 times. Table 2 shows the statistical test results. Table 3 shows the model complexity statistics.

**TABLE 2:** Average performance comparison between different models.

**Model**	**Accuracy (mean ± std)**	**Sensitivity (mean ± std)**	**Specificity (mean ± std)**
Pretrained ResNet-18(ours)	**0.750 ± 0.02**	0.707 ± 0.09	**0.759 ± 0.06**
Pretrained ResNet-34(ours)	0.725 ± 0.01	**0.708 ± 0.08**	0.719 ± 0.11
No-pretrained ResNet-18	0.696 ± 0.01	0.689 ± 0.09	0.707 ± 0.07
No-pretrained ResNet-34	0.690 ± 0.04	0.653 ± 0.15	0.706 ± 0.09
3D ResNet-18	0.669 ± 0.02	0.694 ± 0.06	0.661 ± 0.06
3D ResNet-34	0.689 ± 0.04	0.701 ± 0.07	0.657 ± 0.07

**TABLE 3:** Complexity comparison between different models.

**Model**	**Parameters**	**Training Time(s/epoch)**
ResNet-18(ours)	**11,475,330**	**5**
ResNet-34(ours)	21,583,490	6
3D ResNet-18	33,161,986	16
3D ResNet-34	63,471,618	23

The experimental results show that our model was better than traditional 3D CNNs and non-pretrained models, as the best accuracy, sensitivity and specificity of our models on the validation dataset was 0.75, 0.707 and 0.759 when the depth of our model was 18 layers. The performance of our pretrained model was improved comparing with the 3D baseline and no-pretrained models, it indicates that both the compression block and pretrained datasets were beneficial for performance and generalization ability improvement. When we applied the compression block, the sample space of the image was effectively reduced, and the intrinsic features of images were easier to extract. Table 2 demonstrates that our model needs fewer parameters and less training time.

## Discussion

In this paper, we used deep learning models to automatically process WMH segmentation T2-FLAIR images to distinguish NMOSD from MS automatically, and none of the approaches had manual interventions. Previous studies based on 3D deep learning models focused on diseases with large data sets and concentrated lesions, for example, AD and tumors. Using 3D-CNN to process 3D T2-FLAIR images is indeed in line with people’s intuition. However, since 3D-CNN increases the number of parameters, the model will be low learning efficiency on the training data when the data sets is limited. Especially, the overlapping lesion increase the difficulty for training the model, which makes the generalization ability of the traditional 3D models is weak. The experimental result also shows that the traditional 3D CNN model lacks the learning capacity for NMOSD and MS MRI T2-FLAIR images. There are some reasons for those results:i) The data set size was limited because of the disease’s rareness, which means the model did not feed in abundant data to be generalized. ii) The sample space was too large due to the wide distribution of lesions. iii) Only about half of the NMOSD patients have typical brain lesions, and the brain lesion distribution of NMOSD and MS have overlap in lesion localization (Cacciaguerra et al., 2019). Transfer learning is a widely used technique that allows the model to have a better initial parameter, which will enhance the performance of the model, especially for dealing with small sample data. Currently, there is no 3D pre-trained model available to apply to NMOSD and MS T2-FLAIR images. In Experiment 2, our model compressed the 3D data and then did fine-tuning to effectively improve the performance, which also proved that models pre-trained on ImageNet datasets have better generalization ability for medical images. Our model effectively reduced the sample space dimension and reduced parameter amount, making the model much more easily to train and increase the generalization ability of our model.

This study has some limitations. First, distinguishing NMOSD from MS by automatic WMLs segmentation is a great challenge because of their variability and scattered spatial distribution. In this study, three high error rate cases disagreed with the necessary diagnostic categories due to imprecise segmentation. The outliers had lesions that were part of a confluence of lesions or were located very close to the ventricles, which are extremely difficult for automatic quantification, and semimanual lesion outline correction will be conducted in our next study. Second, the AQP4-IgG seronegative NMOSD patients were not excluded from this study to avoid introducing demographic information bias. However, it is a clinical challenge to distinguish AQP4-IgG seronegative NMOSD from atypical MS. The mean proportion of agreement for diagnosing the two diseases was low among expert clinicians (Po = 0.5) (Wingerchuk et al., 2015), which means the AQP4-IgG seronegative NMOSD subjects might increase the difficulties in the classification of these two diseases. Besides, MS subjects with lesions mimicking stroke were also likely to be mistaken as NMOSD. Third, the prevalence of NMOSD is approximately 0.52–4.4 per 100,000 individuals (Wingerchuk et al., 2007); thus, only a small sample size was recruited in this pilot study, bringing challenges for deep learning models.

## Data Availability Statement

The datasets presented in this article are not readily available because we do not share them directly due to the ethics of the clinical study. Requests to access the datasets should be directed to CG, guocj@jlu.edu.cn.

## Ethics Statement

The studies involving human participants were reviewed and approved by the local ethics committee of the First Hospital of Jilin University, China (2015-133). The patients/participants provided their written informed consent to participate in this study.

## Author Contributions

ZW, ZY, and CG performed study conception and design. ZW, YaoW, and YanW performed the study. HZ, YL, and LS analyzed the data. YaoW and CG drafting of the manuscript. YanW and CG performed critical revision. All authors contributed to the article and approved the submitted version.

## Conflict of Interest

LS is the director of BrainNow Medical Technology Limited. YL is an employee of BrainNow Medical Technology Limited, which developed AccuBrain^®^ used in this paper.

## References

[B1] Abrigo J. Shi L. Luo Y. Chen Q. Chu W. C. W. Mok V. C. T. (2019). Standardization of hippocampus volumetry using automated brain structure volumetry tool for an initial Alzheimer’s disease imaging biomarker. *Acta Radiol.* 60 769–776. 10.1177/0284185118795327 30185071

[B2] Bengio Y. Lecun Y. (2007). “Scaling learning algorithms towards AI,” in *Large-Scale Kernel Machines*, Vol. 34 eds Bottou L. Chapelle O. DeCoste D. Weston J. (Cambridge, MA: MIT Press), 1–41.

[B3] Bianchini M. Scarselli F. (2014). On the complexity of neural network classifiers: a comparison between shallow and deep architectures. *IEEE Trans. Neural Netw. Learn. Syst.* 25 1553–1565. 10.1109/tnnls.2013.2293637 25050951

[B4] Cacciaguerra L. Meani A. Mesaros S. Radaelli M. Palace J. Dujmovic-Basuroski I. (2019). Brain and cord imaging features in neuromyelitis optica spectrum disorders. *Ann. Neurol.* 85 371–384. 30635936 10.1002/ana.25411

[B5] Eshaghi A. Wottschel V. Cortese R. (2016). Gray matter MRI differentiates neuromyelitis optica from multiple sclerosis using random forest. *Neurology* 87 2463–2470. 10.1212/wnl.0000000000003395 27807185 PMC5177679

[B6] Guo C. Niu K. Luo Y. Shi L. Wang Z. Zhao M. (2019). Intra-scanner and inter-scanner reproducibility of automatic white matter hyperintensities quantification. *Front. Neurosci.* 13:679. 10.3389/fnins.2019.00679 31354406 PMC6635556

[B7] Hara K. Kataoka H. Satoh Y. (2017). “Learning spatio-temporal features with 3d residual networks for action recognition,” in *Proceedings of the International Conference on Computer Vision and Pattern Recognition*, Nashville, TN, 3154–3160.

[B8] Hara K. Kataoka H. Satoh Y. (2018). “Can spatiotemporal 3D CNNs retrace the history of 2D CNNs and ImageNet,” in *Proceedings of the International Conference on Computer Vision and Pattern Recognition*, Salt Lake City, UT, 6546–6555.

[B9] He K. Zhang X. Ren S. (2016). “Deep residual learning for image recognition,” in *Proceedings of the International Conference on Computer Vision and Pattern Recognition*, Las Vegas, NV, 770–778.

[B10] Ioffe S. Szegedy C. (2015). “Batch normalization: accelerating deep network training by reducing internal covariate shift,” in *Proceedings of the International Conference on Machine Learning*, Vienna, 448–456.

[B11] Jacob A. Hutchinson M. Elsone L. (2012). Does natalizumab therapy worsen neuromyelitis optica? *Neurology* 79 1065–1066. 10.1212/wnl.0b013e31826845fe 22914835

[B12] Kim H. J. Paul F. Lana-Peixoto M. A. (2015). MRI characteristics of neuromyelitis optica spectrum disorder. *Neurology* 84 1165–1173. 25695963 10.1212/WNL.0000000000001367 PMC4371410

[B13] Kim S.-H. Kim W. Li X. F. Jung I. J. Kim H. J. (2012). Does interferon beta treatment exacerbate neuromyelitis optica spectrum disorder? *Mult. Scler. J.* 18 1480–1483. 10.1177/1352458512439439 22354738

[B14] Kurtzke J. F. (1983). Rating neurologic impairment in multiple sclerosis: an expanded disability status scale (EDSS). *Neurology* 33 1444–1452. 10.1212/wnl.33.11.1444 6685237

[B15] Liang S. Zhang R. Liang D. Song T. Ai T. Xia C. (2018). Multimodal 3D densenet for IDH genotype prediction in gliomas. *Genes* 9:382. 10.3390/genes9080382 30061525 PMC6115744

[B16] Litjens G. Kooi T. Bejnordi B. E. (2017). A survey on deep learning in medical image analysis. *Med. Image Anal.* 42 60–88. 28778026 10.1016/j.media.2017.07.005

[B17] Luo Y. (2017). Automate the quantitative calculation method of subregion brain atrophy. China Patent NO CN107103612B. Shanghai: China Patent and Trademark Office.

[B18] Milletari F. Navab N. Ahmadi S. (2016). “V-net: fully convolutional neural networks for volumetric medical image segmentation,” in *Proceeding of the International Conference on 3D Vision (3DV)*, Stanford, CA, 565–571.

[B19] Montufar G. Pascanu R. Cho K. (2014). “On the number of linear regions of deep neural networks,” in *Proceedings of the International Conference on Neural Information Processing Systems*, Tallinn, 2924–2932.

[B20] Nie D. Zhang H. Adeli E. Liu L. Shen D. (2016). 3D deep learning for multi-modal imaging-guided survival time prediction of brain tumor patients. *Med. Image Comput. Comput. Assist. Interv.* 9901 212–220. 10.1007/978-3-319-46723-8_25 28149967 PMC5278791

[B21] Pan S. J. Yang Q. (2010). A survey on transfer learning. *IEEE Trans. Knowl. Data Eng.* 22 1345–1359.

[B22] Payan A. Montana G. (2015). “Predicting Alzheimer’s disease: a neuroimaging study with 3D convolutional neural networks,” in *Proceedings of the International Conference on Pattern Recognition Applications and Methods*, Prague, 355–362.

[B23] Polman C. H. Reingold S. C. Banwell B. (2011). Diagnostic criteria for multiple sclerosis: 2010 revisions to the McDonald criteria. *Ann. Neurol.* 69 292–302. 21387374 10.1002/ana.22366 PMC3084507

[B24] Rastghalam R. Pourghassem H. (2016). Breast cancer detection using MRF-based probable texture feature and decision-level fusion-based classification using HMM on thermography images. *Pattern Recognit.* 51 176–186. 10.1016/j.patcog.2015.09.009

[B25] Ronneberger O. Fischer P. Brox T. (2015). “U-net: convolutional networks for biomedical image segmentation,” in *Proceedings of the International Conference on Medical Image Computing and Computer-Assisted Intervention*, Munich, 234–241.

[B26] Shen W. Zhou M. Yang F. Yang C. Tian J. (2015). Multi-scale convolutional neural networks for lung nodule classification. *Inf. Process. Med. Imaging* 24 588–599. 10.1007/978-3-319-19992-4_46 26221705

[B27] Shi L. Wang D. Liu S. Pu Y. Wang Y. Chu W. C. W. (2013). Automated quantification of white matter lesion in magnetic resonance imaging of patients with acute infarction. *J. Neurosci. Methods* 213 138–146. 10.1016/j.jneumeth.2012.12.014 23261771

[B28] Srivastava N. Hinton G. E. Krizhevsky A. Sutskever I. Salakhutdinov R. (2014). Dropout: a simple way to prevent neural networks from overfitting. *J. Mach. Learn. Res.* 15 1929–1958.

[B29] Tajbakhsh N. Shin J. Y. Gurudu S. R. Todd Hurst R. Kendall C. B. Gotway M. B. (2016). Convolutional neural networks for medical image analysis: full training or fine tuning? *IEEE Trans. Med. Imaging* 35 1299–1312. 10.1109/tmi.2016.2535302 26978662

[B30] Thompson A. J. Banwell B. L. Barkhof F. (2018). Diagnosis of multiple sclerosis: 2017 revisions of the McDonald criteria. *Lancet Neurol.* 17 162–173. 29275977 10.1016/S1474-4422(17)30470-2

[B31] Wang C. Zhao L. Luo Y. Liu J. Miao P. Shi L. (2019). Structural covariance in subcortical stroke patients measured by automated MRI-based volumetry. *Neuroimage Clin.* 22:101682. 10.1016/j.nicl.2019.101682 30710874 PMC6357849

[B32] Wang H. Shen Y. Wang S. Xiao T. Deng L. Wang X. (2019). Ensemble of 3D densely connected convolutional network for diagnosis of mild cognitive impairment and Alzheimer’s disease. *Neurocomputing* 333 145–156. 10.1016/j.neucom.2018.12.018

[B33] Wernick M. N. Yang Y. Brankov J. G. (2010). Machine learning in medical imaging. *IEEE Signal Process. Mag.* 27 25–38. 25382956 10.1109/MSP.2010.936730 PMC4220564

[B34] Wingerchuk D. M. Banwell B. Bennett J. L. Cabre P. Carroll W. Chitnis T. (2015). International consensus diagnostic criteria for neuromyelitis optica spectrum disorders. *Neurology* 85 177–189. 26092914 10.1212/WNL.0000000000001729 PMC4515040

[B35] Wingerchuk D. M. Lennon V. A. Lucchinetti C. F. Pittock S. J. Weinshenker B. G. (2007). The spectrum of neuromyelitis optica. *Lancet Neurol.* 6 805–815. 17706564 10.1016/S1474-4422(07)70216-8

[B36] Zacharaki E. I. Wang S. Chawla S. (2009). Classification of brain tumor type and grade using MRI texture and shape in a machine learning scheme. *Magn. Reson. Med.* 62 1609–1618. 10.1002/mrm.22147 19859947 PMC2863141

